# Marital Stability During the Year After Traumatic Brain Injury in an Ecuadorian Sample: A Repeated-Measures Study

**DOI:** 10.3390/jcm13237169

**Published:** 2024-11-26

**Authors:** Guido Mascialino, Paul B. Perrin, Juan Carlos Arango-Lasprilla, Jack D. Watson, Alberto Rodríguez-Lorenzana, Clara Paz

**Affiliations:** 1 Escuela de Psicología y Educación, Universidad de Las Américas, 170137 Quito, Ecuador; gmascialino@gmail.com; 2 School of Data Science and Department of Psychology, University of Virginia, Charlottesville, VA 22903, USA; 3 Central Virginia Veterans Affairs Health Care System, Richmond, VA 23249, USA; 4 Department of Psychology, Virginia Commonwealth University, Richmond, VA 22904, USA; jcalasprilla@gmail.com (J.C.A.-L.); watsonjd4@vcu.edu (J.D.W.); 5 Departamento de Ciencias de la Salud, Universidad Pública de Navarra, 31006 Pamplona, Spain; alberto.rodriguez@unavarra.es; 6 Grupo Bienestar, Salud y Sociedad, Escuela de Psicología y Educación, Universidad de Las Américas, 170137 Quito, Ecuador; clara.paz@udla.edu.ec

**Keywords:** traumatic brain injury, marital stability, repeated-measures study, trajectories, Ecuador

## Abstract

**Background:** Traumatic brain injury (TBI) is a major cause of death and disability worldwide and often leads to long-lasting emotional, physical, and cognitive changes and results in reduced functioning across multiple domains. These changes often lead to strain in marital relationships as the uninjured spouse grapples with adapting to the changes in their partner. **Aims:** The purpose of this study was to examine the probability of marital stability after TBI at 6 and 12 months following injury (i.e., probability trajectory across those two time points), as well as predictors of that probability trajectory. **Methods:** The study design was repeated-measures and observational. Patient recruitment and follow-up took place from January 2018 to March 2020 in Quito, Ecuador. Ninety-seven TBI survivors were recruited while hospitalized in the neurosurgery unit of Hospital Eugenio Espejo, a tertiary care center. Patients were assessed at 6 and 12 months after their injury. Hierarchical linear modeling (HLM) was used to examine baseline predictors of linear marital probability trajectories across 6 and 12 months after injury. A final set of HLMs included each of the previously significant predictors from the first model, time, and the interaction terms between time and the previously significant predictor. **Results:** The first HLM found that marital probability remained stable between 6 and 12 months after TBI. Individuals who were employed at baseline had higher marital probability trajectories than those who had been unemployed. Older individuals had higher marital probability trajectories than younger individuals, and women had higher marital probability trajectories than men. **Conclusions:** This is the first study to examine marital probability trajectories for an Ecuadorian adult population with TBI, and the data are of great value to understanding post-TBI outcomes in the region. These results can inform interventions and support systems to bolster marital resilience in the aftermath of TBI. Further research is warranted to explore the nuances of these relationships and to validate these findings in diverse populations.

## 1. Introduction

Traumatic brain injury (TBI) is a major cause of death and disability worldwide [1]. Per recent estimates, 69 million people a year sustain a TBI globally [2]. However, this likely underrepresents the scope of the problem given that almost half of those with mild injuries do not seek care [3] and TBI is often underdiagnosed by clinicians [4]. TBI often leads to long-lasting emotional, physical, and cognitive changes and results in reduced functioning across multiple domains [5]. Personality changes observed in individuals post-TBI, including emotional lability, impulsivity, aggressiveness, and diminished self-awareness, are often more disabling than cognitive and/or physical changes [6,7,8,9]. In addition, psychiatric disorders such as depression and anxiety are highly prevalent after TBI, which can further impact interpersonal relationships [10]. Depression is especially prevalent, with nearly half of TBI survivors developing major depressive symptoms within the first year, symptoms that often persist over time [11]. Cognitive impairments, such as difficulties with memory, attention, and executive function, are common long-term consequences of TBI and can significantly affect daily functioning. While some symptoms improve over time, others, like issues with information processing speed and complex problem-solving, may persist for years post-injury [12,13]. Functional impairments, including motor and balance issues, often lead to decreased independence in daily activities and employment, with approximately 30% of survivors reporting long-term challenges in these areas [5,14].

Understandably, these sequelae impact interpersonal relationships, including marital status [10,15,16]. These behavioral shifts often lead to strain in marital relationships as the uninjured spouse grapples with adapting to the changes in their partner [10,17,18]. The onset of a caregiving role is frequently necessitated for the uninjured spouse, encapsulating a multidimensional burden encompassing emotional, physical, and financial aspects and generating significant distress for individuals in that role [19].

Previous research has identified a range of predictors influencing marital stability following TBI. Demographic and injury-related variables are commonly observed. Arango-Lasprilla et al. [20] identified factors like younger age, male gender, injury through violence, and high injury severity that predicted marital instability. Notably, among minority groups, greater disability post-injury was paradoxically linked to higher stability, possibly pointing to culturally driven familial cohesion in response to adversity. Stevens et al. [21] examined predictors in military populations, emphasizing that factors like older age, higher education, and prior mental health treatment correlated with greater relationship stability. Additionally, injuries sustained during deployment were associated with an unexpected resilience in relationship outcomes, suggesting that shared trauma experiences in military contexts may foster strengthened bonds post-injury. Kreutzer et al. [22] observed that marital longevity prior to the injury, non-violent injury causes, older age, and lower injury severity were strong protective factors for marital stability. Their research suggests that longer-standing relationships may develop resilience factors that help couples weather the behavioral and emotional impacts of TBI. Additionally, they noted that post-injury employment status, gender, and educational attainment did not significantly affect marital stability, underscoring the primary role of relationship duration and injury context in marital outcomes. Laratta et al. [23] examined marital stability within a two-year follow-up of patients with both TBI and acquired brain injuries (ABI) and found that factors like education and religious commitment provided some protective effects against marital dissatisfaction, especially in the presence of cognitive or physical impairments. This emphasizes the importance of both personal and cultural dimensions in marital stability. Along these lines, Blais and Boisvert [24] reviewed the psychological variables that affect marital adjustments by couples post-TBI, underscoring that cognitive changes in the injured partner, combined with altered familial roles, were central to marital strain. Their findings suggest that fostering adaptive coping strategies within couples can improve relational resilience.

Hammond et al. [25] extended these findings by tracking marital stability over a 10-year period post-injury, concluding that stability was highest among older individuals, women, and those without histories of substance abuse. Substance abuse appeared to exacerbate post-TBI marital challenges, potentially through increased financial and emotional strain. Additionally, the authors found that marital dissolution was highest within the first year post-injury, a critical period for interventions that may stabilize and support marriages under acute stress. Ponsford et al. [13] also tracked patients over 10 years post-injury, noting that marital status remained stable in many cases; however, approximately 30% of couples faced relationship difficulties. This study highlighted that persistent symptoms like cognitive and emotional challenges, despite otherwise stable daily functioning, were significant sources of marital strain even years after the initial injury.

Finally, Van den Broek et al. [10] conducted a systematic review that revealed a multifaceted array of factors impacting both relationship quality and stability post-TBI. The authors categorized these into six domains, including injury severity, personality changes, communication patterns, social dependence, environmental context (e.g., presence of children), and individual coping strategies. Notably, personality changes in the injured partner and a lack of communication emerged as significant disruptors of marital quality, often leading to what is termed “ambiguous loss”, where the uninjured partner may feel they are living with a “different person” post-injury. This complex dynamic highlights the need for targeted therapeutic interventions that address communication and coping strategies, offering support to both partners in the adaptation process. Together, these studies illustrate the critical roles of pre-injury characteristics, injury-specific factors, and socio-demographic contexts in shaping marital outcomes after TBI. Importantly, several factors—such as coping skills, social support, and socioeconomic status—are modifiable, highlighting intervention opportunities to bolster marital resilience.

Despite the considerable literature available on the effect of TBI on marriage, several gaps in the literature exist and need to be addressed. The vast majority of studies on TBI have taken place in high-income countries (HICs), while data for low- and middle-income countries (LMICs) are limited or outright absent [26]. This is particularly troublesome given that LMICs have approximately three-fold higher adjusted estimates of TBI cases than HICs [2]. Furthermore, social safety nets are uncommon or inadequate in many LMICs, meaning the stakes are considerably higher for TBI survivors to preserve interpersonal relationships which act as essential sources of support, marriage being the most significant one.

One of the regions in which TBI sequelae are disproportionately felt is Latin America, where the incidence of TBI is 163 per 100,000, compared to the world average of 106 per 100,000, and where 36% of the of the population lives below the poverty line [27]. In spite of these disparities, TBI research in this region is limited or, in the case of some areas, non-existent altogether [28]. For instance, research about TBI in Ecuador is scarce. To date, only two studies with longitudinal designs have been conducted in Ecuador [29,30]. However, the focus of these has been primarily on medical outcomes of TBI, leaving a significant gap in the literature regarding psychosocial outcomes of this condition, such as its impact on marital status and stability. 

Successfully modeling recovery patterns can inform treatment and policy that move the patient along by addressing the right needs at specific time points. As a result, exploring TBI–marital status probability trajectories serves both theoretical and clinical aims. Given the aforementioned gaps in prior research, the aim of this study is to examine the marital stability of Ecuadorian individuals post-TBI. Our specific research question is the following: “What is the trajectory of marital stability among Ecuadorian adults following TBI at 6 and 12 months post-injury, and what baseline factors predict these stability outcomes”? Because marital stability post-TBI has not been studied in similar Latin-American regions, it was not clear what results this study would yield. However, it was possible that predictors found in other more studied areas, such as age, gender, and injury severity, would also prove significant in our sample.

## 2. Methods

### 2.1. Study Design

The current study was observational and repeated-measures in nature. Patients were assessed at 6 and 12 months post-injury.

### 2.2. Participants 

Individuals with TBI were recruited in the Hospital Eugenio Espejo in Quito, Ecuador, a tertiary care hospital with 20 medical specialties, considered to be a referral facility for treating neurological insults and diseases at a national level. Patient recruitment and follow-up took place from January 2018 to March 2020. The proposed inclusion criteria involved individuals who had sustained a TBI “defined as an alteration in brain function, or other evidence of brain pathology, caused by an external force” [31]. Subjects with TBI met the following criteria: (a) be at least 18 years of age at the time of injury; (b) be admitted to the hospital for acute care within 72 h of the TBI; (c) be able to understand and sign the informed consent form. No additional exclusion criteria were considered. Ninety-seven participants were recruited while hospitalized in the neurosurgery unit. Sociodemographic characteristics of the sample at three data collection points (discharge, 6 months, 12 months) can be seen in Table 1. Patients were approached once medically stable and then assessed with the Galveston Amnesia Orientation Test. A score above 75 for two consecutive days was considered evidence of being outside posttraumatic amnesia. At that point, patients were invited to participate in the study by first hearing an explanation of its purpose, methodology, and commitment, and then being asked to repeat the information presented to ascertain their comprehension. Informed consent was obtained from patients or their family members if they could not demonstrate capacity. Follow-up assessments at 6 and 12 months were conducted by telephone or in person depending on the availability of the participant. The study was approved by the ethics committee of the Eugenio Espejo Hospital.

### 2.3. Instruments

A sociodemographic questionnaire was developed to obtain information about age, gender, education, residence (rural vs. urban), employment, and partnership status (married/civil union vs. non-partnered). Options for the latter included single, separated, divorced, and widowed. Clinical variables were obtained from medical records, including Glasgow Coma Scale at time of emergency room admission. Patients were contacted at 6 and 12 months either in person or by phone and once again asked to answer questions about the aforementioned variables. 

### 2.4. Data Analysis 

Hierarchical linear modeling (HLM) was used to examine baseline predictors of linear marital probability trajectories across 6 and 12 months after injury. Full information maximum likelihood (FIML) estimation was used to account for missing data. Predictors were entered simultaneously as fixed effects into an HLM after being centered or given a reference point of 0, along with time. The first HLM examined whether marital status probability trajectories across the two follow-up time points could be predicted by the demographic and injury characteristics of time (coded as 0 [6 months] or 1 [12 months]), gender (0 = woman, 1 = man), urban vs. rural (0 = urban, 1 = rural), employment status (0 = unemployed, 1 = employed), age, education level, Glasgow Coma Scale (GCS) score, and family income. Age, education, GCS, and family income were entered as continuous variables. Marital status at baseline was not included in the model to prevent near singularity between it as a predictor and an outcome which would account for the vast majority of outcome trajectory variance. To test potential differential effects of the predictors over time (e.g., differences in slope as a function of the predictor), a final set of HLMs included each of the previously significant predictors from the first model, time, and the interaction terms between time and the previously significant predictor. Finally, the data analysis method enabled the retention of participants who were lost to follow-up by employing full information maximum likelihood estimation, ensuring the results remained largely unaffected.

## 3. Results

### 3.1. Sample Characteristics

The repeated-measures study sample included individuals at three time points: discharge (n = 97), 6 months post-discharge (n = 87), and 12 months post-discharge (n = 58). Participants being lost to follow-up was due to being unable to contact participants in spite of our best efforts. Across the full sample, at injury, 6 months, and 12 months, 50.6%, 46.0%, and 44.8% were partnered, respectively. The sample was predominantly male across all time points, with men comprising 92.7% at discharge and 94.7% by 12 months. Urban residence was more common, with around 61.7% of participants living in urban areas at discharge, maintaining a similar proportion over time. Employment status increased slightly, with 82% employed at discharge and 85.4% by 12 months. The mean age of participants remained stable, averaging 35.3–36.3 years, while educational attainment and Glasgow Coma Scale (GCS) scores showed minimal variation. Family income demonstrated some fluctuation, with a slight increase noted at 12 months.

### 3.2. First Model

**Figure 1:**
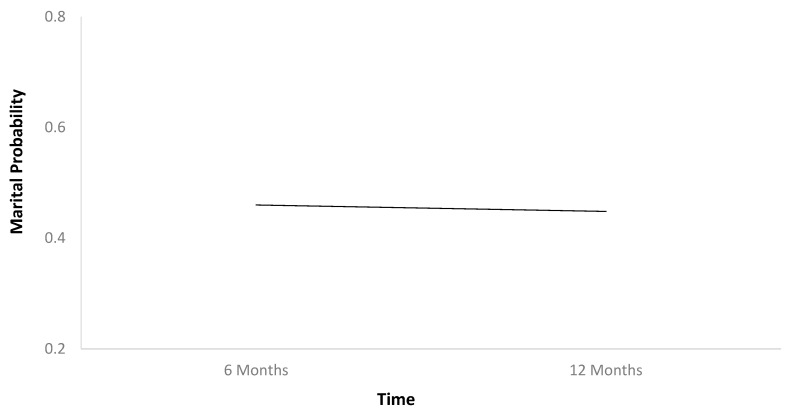
Marital probability over time.

**Figure 2:**
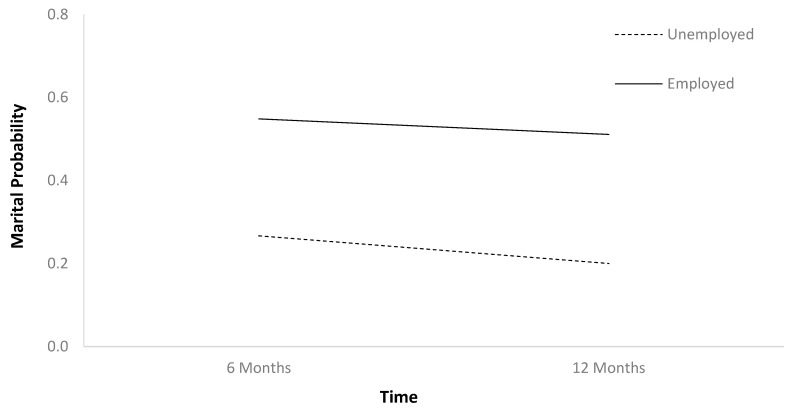
Main effect of employment on marital probability.

**Figure 3:**
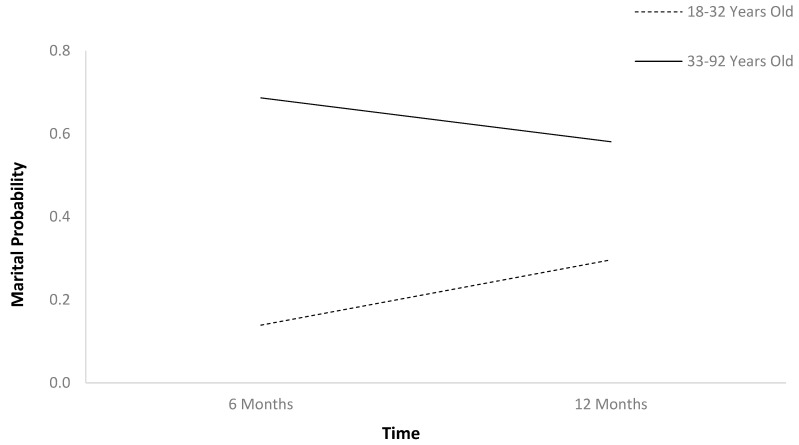
Main effect of age on marital probability.

**Figure 4:**
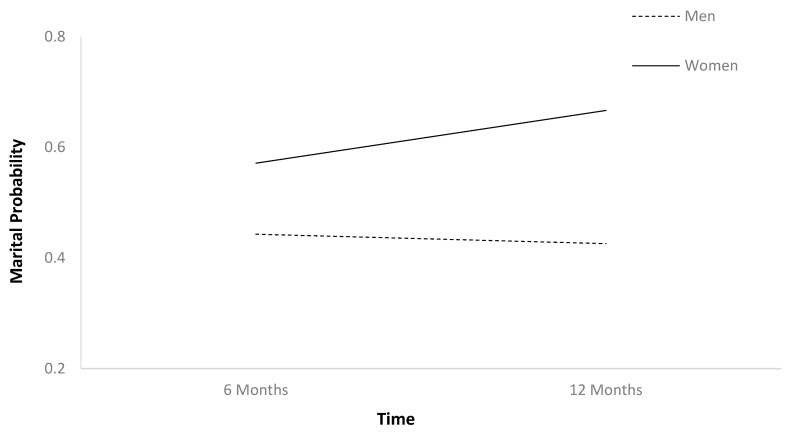
Main effect of gender on marital probability.

The first HLM (Table 2) found that marital probability remained stable between 6 and 12 months after TBI (Figure 1). In Table 2, b-weights are the parameter estimates of the predictor’s statistical effect on relationship status trajectories over time. The respective *p*-values reflect whether these parameter estimates are statistically significant (if less than 0.05), or in other words, whether these baseline variables statistically predict the probability of relationship status holistically across the two time points. Individuals who were employed at baseline had higher marital probability trajectories than those who were unemployed (Figure 2). Older individuals had higher marital probability trajectories than younger individuals (Figure 3), and women had higher marital probability trajectories than men (Figure 4).

### 3.3. Models with Time Interactions

Three follow-up HLMs examined whether the above statistically significant main effects had a differential effect on marital probability trajectories as a function of time (e.g., difference in slope; Table 3). There were no significant interaction terms for time × employment, time × age, or time × gender, suggesting there was no differential change in marital probability between 6 and 12 months after TBI for these variables.

## 4. Discussion

The purpose of this study was to examine the probability of marital stability after TBI at 6 and 12 months following injury (i.e., probability trajectory across those two time points), as well as predictors of that probability trajectory. The results revealed employment at baseline predicted higher marriage probability trajectories than unemployment as baseline. Older subjects also had higher marriage probability trajectories than younger individuals, and women had higher marriage probability trajectories than men. However, the gender effect is based on a small subsample of women vs. a larger subsample of men; as a result, future studies with a more representative sample by gender should replicate these findings. Marital probability remained the same between 6 and 12 months after TBI. No interaction effects with time were noted for these predictors. 

The results of the current study indicating that older age is associated with marital stability are consistent with prior research conducted in other regions. Arango-Lasprilla et al. [20], examining marital stability two years post-TBI, found that older age predicted stability, suggesting that age contributes resilience to long-term relationship dynamics following TBI. Similarly, in a study looking at marital stability two years post-TBI, Kreutzer et al. [22] found that older age at injury predicted stability in a sample of 122 subjects 2.5 to 8 years post-TBI. In a study of 357 active duty service members and veterans, Stevens et al. [21] also found that age predicted marital stability. Vanderploeg et al. [32] found the same result, age as a predictor of marital stability, in a larger sample of veterans (n = 626). The association between age and stability appears to hold for as long as 10 years post-injury, as Hammond et al. [25] found in a TBI model systems study with a large sample of 1423 subjects. According to Wood and Yurdakul [33], the effect of age on relationship stability may be moderated by relationship length, suggesting that the protective effects of age could be attributed to longer pre-injury relationships.

The present study also found employment status to be a predictor of marital stability, aligning with findings from Forslund et al. [34], who observed that employment at the time of injury predicted marital stability at various follow-up points up to five years post-injury in a sample of 105 Norwegian TBI patients. Arango-Lasprilla et al. [20] found that post-injury employment, rather than employment at the time of injury, influenced marital status, suggesting that continued work engagement post-TBI may help preserve relational stability by providing a structured routine and mitigating financial stress. Additionally, Vanderploeg et al. [32] found that individuals employed full-time post-injury had a greater likelihood of being married (OR = 1.89) in a sample including individuals up to 8 years post injury, indicating that economic stability and social engagement through work might play significant roles in relationship maintenance post-TBI. Van den Broek et al. [10] also underscored the importance of employment as a stabilizing factor in marriage, particularly in settings where socioeconomic resources are limited. 

Findings related to gender and marital stability have shown some variability in prior research. In the current study, women demonstrated greater marital stability than men at both six and twelve months post-injury. Arango-Lasprilla et al. [20] found similar results to the current study: the odds of marital instability were 1.95 times greater for males than for females. Hammond et al. [25] also found that being female predicted greater marital stability at 10 years post-injury. However, Kreutzer et al. [22] and Forslund et al. [34] did not find gender differences in marital stability, suggesting that while gender may be a factor, its impact could vary based on sample characteristics or cultural context.

Finally, the results of this study should be interpreted in the cultural context in which it took place. Ecuador is a predominantly Catholic country, with 74.8% of the population identifying as such [35]. Marriage in Ecuador reflects a combination of traditional values and deep-rooted gender inequalities, often shaped by the cultural constructs of *machismo* and *marianismo*. These norms prescribe distinct roles for men and women, positioning men as authoritative heads of families while emphasizing obedience and subservience in women [36,37]. Moreover, economic and property rights within marriage highlight gender inequality, with married women often having limited ownership and decision-making power in family assets, which reinforces economic dependence on male partners [38]. Although some progress in gender equality is emerging through legal reforms, these traditional beliefs and structures continue to influence marriage dynamics, limiting women’s autonomy in various aspects of family life and social standing [39].

In addition, Ecuador provides limited government support for individuals recovering from traumatic brain injuries, largely due to resource constraints in the healthcare system. Studies indicate that rehabilitation services are often insufficient, leaving families with much of the care burden [40]. Some government-led initiatives offer basic healthcare coverage; however, accessibility and quality of care vary significantly, especially for rural populations [41]. Consequently, family members play a critical role in care and rehabilitation, often receiving minimal guidance or resources to manage the prolonged recovery process effectively. It is likely that the results of this study were influenced by these cultural forces, particularly with regard to gender and employment status.

Long-term impacts of TBI on relationships are also most likely affected by psychosocial variables which were not considered in this study, such as caregiver burden, the presence of social support networks, and marital dynamics [21,42,43]. Manskow et al. [42] observed that higher caregiver burden and social isolation could jeopardize relationship stability over time, underscoring the importance of interventions aimed at reducing burden through support networks. Similarly, Van den Broek et al. [10] highlighted how reliance on a partner for emotional support can be protective for the TBI survivor, while the burden on the caregiving partner can increase relational strain, especially in cases where role shifts significantly alter the dynamics of the relationship. Lastly, Kreutzer et al. [43] focused on the dynamics within couples facing varying TBI severities, noting that while marriage stability remained high, significant marital distress was common, with half of the injured and uninjured partners reporting dissatisfaction. This study underscores the distinction between stability and satisfaction, suggesting that some couples may remain together despite considerable relational challenges due to external factors, including financial dependency or caregiving obligations.

In addition to lacking a wider range of psychosocial variables mentioned above, this study presents with other limitations. Repeated-measures research is a complex affair under ideal conditions and even more so when confronted with the realities of LMICs. Patients were lost to follow-up despite the research team’s best efforts. Nevertheless, the data analytic approach allowed for retention of participants lost to follow-up due to the use of full information maximum likelihood estimation without gravely affecting the results. Another study limitation is that it took place in only one hospital and region, thereby limiting the generalizability of results. These results should not be generalized to other areas of Ecuador or Latin America. As such, future work should focus on multi-center efforts to best represent the national and regional makeup. The present study’s shorter follow-up of only one year also limits generalization, as this time frame may be insufficient to detect meaningful changes in long-term relationship stability, particularly given that many previous studies have tracked stability for five or more years post-injury. Also, the analysis did not include other variables that could be related to marital probability trajectories, such as history of alcohol and/or substance abuse, premorbid cognitive and behavioral functioning, social support, insurance, and pre-injury occupational prestige (e.g., blue collar vs. white collar). As these factors have been shown in studies such as Wood and Yurdakul [33] (1997) to impact relationships, future research would benefit from including them to better understand their role in relationship stability post-TBI. Lastly, this study focused on sociodemographic and clinical predictors that cannot be easily modified, if at all. Future studies should focus on modifiable factors such as time spent in inpatient and outpatient rehabilitation, type of services received, and pharmacological interventions for cognitive and/or behavioral problems, which have been shown to affect post-injury relationship quality and could potentially inform intervention programs. 

## 5. Conclusions

Despite these limitations, the current study had strong internal validity, finding that a number of baseline variables predicted marital probabilities over time. While causality cannot be determined due to the lack of an experimental design, these associations underscore the ability of the study’s design and power to illuminate these predictive effects over time. This is the first study to examine marital probability trajectories for an Ecuadorian adult population with TBI, and the data are of great value to understanding post-TBI outcomes in the region. Older age, being a woman, and being employed at the time of the injury appear to be protective factors. These results can inform interventions and support systems to bolster marital resilience in the aftermath of TBI. Further research is warranted to explore the nuances of these relationships and to validate these findings across diverse settings and populations.

## Figures and Tables

**Table 1:** Demographic and injury variables at discharge, 6 months, and 12 months.

	Discharge (n = 97)	6 Months (n = 87)	12 Months (n = 58)
Marital status			
Yes	45 (50.5%)	40 (46%)	26 (44.8%)
No	44 (49.5%)	47 (54%)	32 (55.2%)
Gender			
Women	7 (7.3%)	7 (8%)	3 (5.3%)
Men	89(92.7%)	79 (92%)	54 (94.7%)
Residence			
Rural	33 (38.3%)	29 (38%)	22 (41.5%)
Urban	53(61.7%)	48 (62%)	31 (58.5%)
Employment			
Yes	70 (82%)	46 (58%)	41 (85.4%)
No	16 (18%)	33 (42%)	7 (14.6%)
Age	35.3 (13.3)	36.3 (13.4)	35.3 (14.2)
Education	9.5 (3.9)	9.5 (3.6)	10 (4.2)
GCS	11.9 (3.9)	11.9 (3.9)	11.8 (3.9)
Family Income	758 (672)	727 (635)	847 (683)

**Table 2:** Demographic and injury predictors of relationship status trajectories across 6 and 12 months.

Predictor	b-Weight	*SE*	*p*-Value	95% Confidence Interval	
				Lower Bound	Upper Bound
Intercept	0.67	0.19	<0.001	0.28	1.05
Time	−0.03	0.07	0.641	−0.17	0.10
Gender (0 = woman, 1 = man)	−0.44	0.21	0.043	−0.87	−0.01
Rural vs. Urban (0 = urban, 1 = rural)	−0.10	0.10	0.318	−0.31	0.10
Employed (0 = unemployed, 1 = employed)	0.31	0.15	0.038	0.02	0.61
Age	0.01	0.00	0.023	0.00	0.02
Education	−0.01	0.02	0.573	−0.04	0.02
GCS	0.02	0.01	0.223	−0.01	0.04
Family Income	0.00	0.00	0.334	−0.00	0.00

**Table 3:** Previously significant predictors and time interactions on marital probability across 6 and 12 months after TBI.

Predictor	b-Weight	*SE*	*p*-Value	95% Confidence Interval	
Employment HLM				Lower Bound	Upper Bound
Intercept	0.26	0.12	0.038	0.01	0.51
Time	−0.09	0.14	0.501	−0.37	0.18
Employment (0 = unemployed, 1 = employed)	0.78	0.14	0.047	0.00	0.55
Time × Employed	0.07	0.15	0.629	−0.23	0.38
Age HLM					
Intercept	0.44	0.05	<0.001	0.35	0.54
Time	−0.01	0.06	0.884	−0.12	0.11
Age	0.01	0.00	<0.001	0.01	0.02
Time × Age	−0.00	0.00	0.489	−0.01	0.01
Gender HLM					
Intercept	0.57	0.19	0.003	0.20	0.94
Time	−0.17	0.24	0.468	−0.64	0.30
Gender (0 = woman, 1 = man)	−0.14	0.20	0.481	−0.53	0.25
Time × Gender	0.16	0.24	0.505	−0.32	0.65

## Data Availability

The original contributions presented in the study are included in the article, further inquiries can be directed to the corresponding authors.
